# Fetal alcohol spectrum disorders and their transmission through genetic and epigenetic mechanisms

**DOI:** 10.3389/fgene.2014.00154

**Published:** 2014-06-02

**Authors:** Edward A. Mead, Dipak K. Sarkar

**Affiliations:** Rutgers Endocrine Program, Department of Animal Sciences, Rutgers, The State University of New Jersey,New Brunswick, NJ, USA

**Keywords:** fetal alcohol, FASD, HPA axis, proopiomelanocortin, transgenerational epigenetic

## Abstract

Fetal alcohol spectrum disorders (FASD) are a group of related conditions that arise from prenatal exposure to maternal consumption of the teratogen, ethanol. It has been estimated that roughly 1% of children in the US suffer from FASD (Sampson etal., 1997), though in some world populations, such as inhabitants of some poorer regions of South Africa, the rate can climb to as high as 20% (May etal., 2013). FASD are the largest cause of mental retardation in U.S. neonates, and ironically, are entirely preventable. FASD have been linked to major changes in the hypothalamic-pituitary-adrenal (HPA) axis, resulting in lifelong impairments through mental disorders, retardation, and sensitivity to stress. FASD are linked to an impaired immune system which consequently leads to an elevated risk of cancer and other diseases. FASD arise from a complex interplay of genetic and epigenetic factors. Here, we review current literature on the topic to tease apart what is known in these areas particularly emphasizing HPA axis dysfunction and how this ties into new studies of transgenerational inheritance in FASD.

## INTRODUCTION

A negative impact from alcohol consumption has been observed since ancient times, leading to cultural prohibitions on alcohol consumption, particularly among women of childbearing years. The ancient Greeks demonstrated some awareness of the risks of alcohol consumption – for example, Plato proposed limiting wine consumption in people under 40 years of age (Abel, 1984). In some ancient societies, such as the Carthaginian Empire, the prohibitions were even written into law (Jones et al., 1973; Abel, 1984). Biblical and Talmudic references suggest that possibly, the ancient Hebrews might have had some awareness that alcohol consumption among fathers and mothers prior to conception could be harmful to the offspring (Abel, 1984). Ancient Vedic writings list prohibitions for drinking by Brahmins (Sharma et al., 2010) suggesting recognition of some negative effects of alcohol, though it is not clear whether an impact on offspring was known. Many sources discussing parental alcohol consumption before modern times focused upon paternal consumption more than maternal (Abel, 1984). With the writings of Francis Bacon in 1627, we see definite concerns about the impact of maternal alcohol consumption on offspring, and in the early 18th century in England, the “gin epidemic” gave rise to commentary that drinking specifically during pregnancy harmed the developing child (Abel, 1984).

Studies in guinea pigs a century ago found that not only did paternal chronic alcohol consumption result in higher mortality rates of offspring, but that the mortality rate was elevated even in the grandchildren of the alcohol-consuming father (Stockard, 1913), giving the first known evidence of a potential multigenerational effect of alcohol exposure. Fetal alcohol syndrome (FAS), the more severe manifestations of the FASD, first began to appear in the medical literature in 1973 (May and Gossage, 2011), and research in the field of fetal alcohol exposure began to take off as the research community launched a coordinated effort into understanding FASD. This led to the recent discovery of a transgenerational impact of fetal alcohol exposure upon stress axis dysfunction. This impact was discovered to be mediated by epigenetic mechanisms carried specifically in the male germline of rats (Govorko et al., 2012).

### DYSREGULATION OF THE HPA AXIS IN FETAL ALCOHOL EXPOSED OFFSPRING

The fetal stage of human life is arguably the most susceptible to harm from alcohol as the fundamental development of organs and pathways occurs in this stage. Not surprisingly, mothers who consume alcohol despite the risks may create severe developmental problems in their offspring. FASD, as its name implies, comprises a wide range of mental, emotional, craniofacial, physiological, and immune disorders which arise from maternal consumption of alcohol. In general, severity of the disorder correlates with the degree of maternal alcohol consumption, though some individuals are more resistant to the effects than others. Individuals with milder forms of FASD may not show any obvious deformities, but may be affected with hyperactivity, depression, anxiety or other disorders that impair quality of life (Schneider et al., 2002). At the other end are individuals with visible deformities, severe retardation, an impaired immune system, impaired metabolic function, and lifelong problems coping with stress, individuals who have FAS (Momino et al., 2012). At the molecular level, many intertwined causal factors contribute to FASD leading to the varied impacts seen among those who suffer. Among the factors, FASD is intimately tied to hyperstress-response and anxiety disorders that are connected to the dysregulation of hypothalamic-pituitary-adrenal (HPA) axis functions.

The HPA axis is a complex neuroendocrine loop maintained by crosstalk between the hypothalamus, pituitary, and the peripheral adrenal glands. The paraventricular nucleus (PVN) of the hypothalamus generates corticotrophin-releasing hormone (CRH) and vasopressin, stimulating the production of the precursor polypeptide proopiomelanocortin (POMC) in the anterior lobe of the pituitary. The breakdown of POMC releases adrenocorticotropic hormone (ACTH) initiating the delivery of glucocorticoids (GCs) to peripheral circulation from the adrenal glands. The primary GC produced in the human adrenal cortex is cortisol (corticosterone in rats). Corticosterone can inhibit the production of POMC in the anterior lobe of the pituitary in rats, creating a feedback loop (Eberwine and Roberts, 1984). GCs reduce inflammatory responses through an immunosuppressive action, and stimulate the sympathetic “fight or flight” response giving a rapid, temporary boost to an organism responding to an environmental threat. The autonomic nervous system complements the stress response, either through “fight or flight” (mentioned above), or by “tend and mend,” an opposing process through the parasympathetic nervous system. ACTH also stimulates the production of catecholamines (CATs) from the adrenal glands. Epinepherine, also known as adrenaline, and its counterpart, norepinephrine (noradrenaline) are CATs released by the adrenal medulla that activate the sympathetic stress response, leading to many of the common physiological symptoms of stress, such as sweating, dry mouth, and a rapid heartbeat. Though the sympathetic stress response increases survival, it comes at a significant cost in terms of metabolism, the immune system, digestion, and other physiological processes, and cannot be maintained indefinitely (reviewed in Rachdaoui and Sarkar, 2013; Wynne and Sarkar, 2013).

β-endorphin is another peptide product of POMC generated through the stress response. β-endorphin is an opioid that can regulate pain, but it also regulates ACTH (another peptide product of POMC; Fratta et al., 1981) and corticotrophin-releasing hormone (CRH; Plotsky, 1986). Hypothalamic β-endorphin is important to the homeostasis of the stress response. CRH and catecholamines stimulate β-endorphin release that suppresses the HPA axis response (Boyadjieva et al., 2009). δ and μ opioid receptors bind central β-endorphin, regulating the autonomic nervous system through the PVN. Levels of pituitary β-endorphin have a smaller role on the autonomic nervous system and are regulated by arginine vasopressin (AVP) and CRH (reviewed by Sarkar et al., 2012).

Proopiomelanocortin neurons in the arcuate nucleus of the hypothalamus play a critical function in regulation of the HPA axis as well as reward pathways and the immune system, through the neuropeptides melanocortin, ACTH, and β-endorphin derived from the POMC precursor polypeptide (Sarkar et al., 2012). POMC neuronal functions were found to be impaired in fetal alcohol-exposed (FAE) rats (Sarkar et al., 2007; Hellemans et al., 2008; Boyadjieva et al., 2009). Recent experiments showed that replacing β-endorphin/POMC-producing cells in FAE rats led to an improvement in stress and immune response in the animals, demonstrating a role for POMC in FASD (Boyadjieva et al., 2009).

## VARIANT ALLELES OF GENES INVOLVED IN DEVELOPMENT, THE HPA AXIS, AND ALCOHOL METABOLISM PLAY A STRONG ROLE IN FASD SUSCEPTIBILITY AND SYMPTOMS

Though studies showing heritable damage from fetal alcohol exposure go back at least a century (Stockard, 1913), it was only with the use of molecular biology approaches during the 1980s that science began to unravel the genetic underpinnings of FASD. At the chromosomal level, structural damage was observed at a high frequency among prenatally exposed individuals in a clinical setting. In one study, 8.75% (7/80) of FASD patients in a genetic screening were determined to have chromosomal abnormalities, typically microduplications or microdeletions (Douzgou et al., 2012). Strong evidence for a genetic component to FASD includes a comparison between mono- and dizygotic twins. Monozygotic twins (having the same genome) show 100% concordance for FASD; that is, if 1 twin has FASD, the other will also have FASD. In comparison, dizygotic twins (having genomes that are moderately different) show only 63% concordance (Streissguth and Dehaene, 1993), showing that even the modest genetic differences of siblings sharing the same environment lead to a significantly different rate of susceptibility to FASD.

It is known that multiple genetic loci are affected by alcohol, with each variant allele interacting in a complex biological pathway with other genes (Johnson et al., 2006). In FASD, this complexity is multiplied as the genes of the mother can impact the fetal environment and expression of genes of the developing fetus, shaping its susceptibility toward fetal alcohol exposure. Maternal RNA and proteins are present in the oocyte to allow for development to occur in the early embryo until it has developed to the Maternal-to-Zygotic transition (MZT) at which point the embryonic cellular processes take over (Schier, 2007). Maternal hormones have also been shown to play an important role in development of an offspring. These examples show that maternal genes (and their products) can have a strong impact on the development of the fetus beyond contribution of genes alone, dubbed the “maternal effect.” By contrast, transmission of a phenotype from the father passed through the father’s sperm (“paternal effect”) is more limited but has been demonstrated (Fitch et al., 1998). In short, altered parental expression of important genes could result in significant vulnerability or resistance of the offspring to ethanol-induced dysregulation in embryonic development even in the absence of those genes in the child, laying down a dysfunctional or more resistant foundation that lasts a lifetime.

A number of genes have also been identified as potentially playing a direct role in FASD. In a study of rhesus monkeys, it was demonstrated that fetal monkeys carrying a short serotonin transporter gene polymorphic region variation (rh5-HTTLPR), an allele with a functional analog in humans, were particularly susceptible to prenatal alcohol exposure during early gestation, leading to sensory disorders (Schneider et al., 2009). Prior studies indicated that this allele was linked to a greater incidence of irritability and stress-responsiveness in monkey offspring subjected to prenatal alcohol exposure (Kraemer et al., 2008).

Genes coding for alcohol dehydrogenase variants have been found to be particularly relevant for FASD, sometimes resulting in varied incidence or severity of FASD. In some cases, they may alter maternal drinking patterns: ADH variants have been linked to alcohol addiction, while other variants may cause mothers to drink less. Alcohol dehydrogenases are found ubiquitously across the kingdoms of living organisms. The products of these genes are involved in converting alcohols (ROH) into aldehydes (R-CHO) and ketones (RCOR’) through reduction of the coenzyme nicotinamide adenine dinucleotide (NAD^+^) into NADH. Humans have six different ADHs, and alcohol dehydrogenase plays an important role in ethanol metabolism and alcohol addiction. Slower metabolizing variants of ADH2 and -3 have been linked to alcoholism in Asian populations (Osier et al., 1999). ADH1B has a strong link to alcoholism (Li et al., 2011). In FASD, it was discovered that maternal variant alleles of ADH1B, ADH1B*2, and ADH1B*3 (both relatively common in African-descended populations) which metabolize alcohol rapidly, led to reduced incidence of FASD in offspring, even those without the gene (Warren and Li, 2005; Jacobson et al., 2006). It is known that ADH1B*3 leads to reduced alcohol intake, so possibly it creates an unpleasant association with elevated aldehyde levels through rapid metabolization of alcohol, or the enzyme could help protect offspring by reducing the peak blood alcohol levels, a critical factor in determining the amount of damage done to fetuses (Jacobson et al., 2006), suggesting that these could be underlying mechanisms for reducing the incidence of FASD. In fetal mice exposed to alcohol, variant genotypes of *aldh2*, an alcohol dehydrogenase gene, have been linked to defects in brain development and vision. This was exacerbated in mice lacking *fancd2*, which plays a downstream role in processing aldehyde metabolites of alcohol (Langevin et al., 2011). In zebrafish, *mars* (a gene involved in alcohol metabolism) null fish were linked to developmental face and brain dysfunction during an embryonic alcohol study (McCarthy and Eberhart, 2014). Other genes impacted by ethanol in an embryonic alcohol study conducted in zebrafish include *hinfp, plk1, foxd1*, and *vangl2*, which have significance in cellular processes such as translation and the cell cycle, and in early development (McCarthy and Eberhart, 2014). Bioinformatic data mined from the results of published literature searches identified from a screen of over 10,000 candidate genes, a subset of 87 genes within the TGF-β, MAPK, and Hedgehog signaling pathways which were likely relevant for FASD. These include *gnas*, and *msx1*, important in apoptosis and cell signaling, *fgfr1-3*, important for embryonic bone development, and *bmp4*, important in myogenesis. Also included were *foxg1b*, *hoxa1*, and *pax6*, important in brain development (Lombard et al., 2007). These genes were examined through pathway, protein-protein and transcription binding analysis and are rich targets for studies into the genetics of FASD. They also fit well with observed phenotypic changes in FASD patients including facial deformities, cardiovascular irregularities, skeletal defects, and brain growth defects (Clarren et al., 1978; Clarren, 1986).

In summary, many genes, either related to alcohol metabolism, or to development and the HPA stress axis, have been identified in relation to FASD. Variants of some of these genes have been shown to contribute to the varied responses seen to fetal alcohol exposure, and it seems likely that other genes also contribute. This may explain in part why the percentage of women who drink during pregnancy is so much higher than the percentage of children who are born with FASD.

## FETAL ALCOHOL EXPOSURE CAUSES EPIGENETIC CHANGES CRITICAL TO FASD

Epigenetics refers to changes in gene expression that do not arise from changes in the underlying DNA sequence. Environmental toxins such as ethanol may impact the expression of genes through altering DNA methylation patterns or modifying histone tails by methylation or acetylation. Epigenetic studies related to FASD are still an emerging field (Haycock, 2009) but have led to the discovery that many symptoms of FASD can be traced back at least in part to aberrant epigenetic marks laid down during gamete production, or during embryonic development under the influence of alcohol. Recent studies have found epigenetic changes due to ethanol that are permanent (Govorko et al., 2012), and they can act broadly across the genome (Kaminen-Ahola et al., 2010). Both DNA methylation and histone modifications, two of the most commonly studied epigenetic mechanisms, can alter the accessibility of DNA to the molecular transcriptional machinery, providing a powerful method for ethanol to create developmental havoc through changing the expression of genes (Renthal and Nestler, 2009).

### DNA METHYLATION

The process of DNA methylation involves the transfer of a methyl group by a DNA methyltransferase (DNMT), utilizing S-adenosyl methionine (SAM), to the C^5^ carbon of a cytosine residue, typically in regions containing strings of CpG dinucleotides (CpG islands; Bestor, 2000). CpG islands (CGIs) are traditionally defined by having an extended stretch of nucleotides (>200 bases), a C/G nucleotide composition above 50%, and an observed CpG dinucleotide content of 65+% (Gardiner-Garden and Frommer, 1987). Roughly 70% of annotated promoters show the presence of CGIs that either contain transcription start sites (TSS) or are near them (Saxonov et al., 2006). In vertebrates, CpGs are in low overall abundance and often methylated, however, CpGs in CGIs are frequently unmethylated (Deaton and Bird, 2011). Approximately half of CGIs in mice and humans are associated with TSS (Illingworth et al., 2010; Deaton and Bird, 2011). The remaining, dubbed “orphan CGIs” are themselves often associated with novel promoters (Illingworth et al., 2010; Maunakea et al., 2010).

Methylation of CpG islands is often regulated during development to control gene expression and the level of methylation correlates with the magnitude of gene inactivation (Doerfler, 1983).

Alcohol may affect DNA methylation through an impact on key methylating enzymes such as DNA methyltransferases (DNMTs). Studies have shown that a downstream metabolite of alcohol breakdown, acetaldehyde, can inhibit DNMT1 (Garro et al., 1991). DNA methylation changes can also occur by altering the interconnected choline, methionine and 5-methyltetrahydrofolate (folate) pathways, which provide key substrates for methylation (Hamid et al., 2009). Alcohol exposure may act through folate (Hidiroglou et al., 1994) and *S*-adenosylmethionine (SAM; Barak et al., 1993; Lu et al., 2000). Alcohol reduces the absorption of folate from the diet, a vitamin that is necessary for carbon-transfer reactions in the methionine–homocysteine pathway, putting limits on DNA methylation (Halsted et al., 2002). Folate deficiency is linked to global DNA hypomethylation (Kim et al., 1997). Choline supplementation reduces FASD symptoms by providing sufficient methyl groups to the methinone–homocysteine pathway (Thomas and Tran, 2012; Bekdash et al., 2013; Wozniak et al., 2013).

Alcohol-mediated aberrant methylation has been documented in at least several developmental genes in alcohol-abusing humans (Bönsch et al., 2006; Hillemacher et al., 2009). In addition, alcohol can act through demethylation (Ponomarev, 2013). DNA methylation changes have been observed to occur during early development. Liu et al. (2009) found that extensive methylation occurred in developmental genes of alcohol-exposed mouse embryos (Liu et al., 2009). Kaminen-Ahola et al. (2010) found that the A^vy^ gene in mice was hypermethylated following alcohol exposure in early fetal development. Prenatal alcohol exposure studies in mice demonstrated DNA methylation changes resulting in neurofacial and growth defects analogous to FASD in humans (Liu et al., 2009). Alcohol exposure alters DNA methylation profiles in mouse embryos at early neurulation and alters methylation of imprinted genes, known to play roles in the cell cycle, growth, apoptosis, cancer, and in a large number of genes associated with olfaction. These results indicate that gene specific and global changes in DNA methylation occur in response to alcohol during fetal development.

Exposures to ethanol and other toxicants during critical windows in development can be particularly damaging and can cause epigenetic changes in gene expression of the brain that impact the brain for the life of the organism (Kobor and Weinberg, 2011; Ungerer et al., 2013; Nestler, 2014). Alcohol is highly damaging before and during very early development, specifically at three events. When gametes are being produced or are maturing, they are vulnerable to alcohol abuse (Haycock, 2009) as demonstrated by several studies (Ouko et al., 2009; Govorko et al., 2012). This is likely because DNA is being actively repackaged during this period. Also germ cells persist for extended periods of time in the body, providing a longer window for the actions of repeated exposures to environmental toxins like ethanol. A second highly vulnerable period occurs after fertilization but before implantation, when the embryo is undergoing rapid developmental changes and is preparing for implantation. The third occurs at gastrulation, when the three germ layers are being defined. As a result, histone marks are being laid down to specify cell types (Shi and Wu, 2009), and the developing embryo is at its most vulnerable to alcohol at this stage (Armant and Saunders, 1996; Haycock, 2009).

Rat-based studies have found that POMC expression is affected by fetal alcohol exposure, leading to the loss of β-endorphin producing neurons and a reduction in POMC expression (Chen et al., 2006; Sarkar et al., 2007; Kuhn and Sarkar, 2008). It has been found that ethanol is able to increase DNA methylation of the promoter region of POMC in fetal pups, changing the epigenetic markings to downregulate POMC expression (Govorko et al., 2012). This is important as POMC neurons, located in the hypothalamus, are critical for bringing stress homeostasis. The perturbation of the HPA axis is a direct cause of many of the symptoms associated with FASD including deficient stress response, depression, anxiety, and impaired immunity (Sarkar, 1996; Pritchard et al., 2002; Raffin-sanson et al., 2003; Sarkar et al., 2012; Rachdaoui and Sarkar, 2013).

In short, DNA methylation has been found to be a strong factor in the incidence of FASD. Alcohol acts through several routes to affect DNA methylation, and seems to be particularly damaging at specific stages of embryonic development resulting in significant changes in methylation that can last a lifetime.

### HISTONE MODIFICATION

Histone modifications are other major biological processes by which epigenetic modification of gene expression occurs. DNA is wound around histones, affecting its ability to interact with transcriptional machinery. Tight packaging results in less interaction. Covalent modifications to the tails of histones 3 and 4 (H3 and H4) are commonly studied. However, H3 and H4 are not the only histones that can be epigenetically modified. For H3 and H4, heterochromatin, the silenced state, is associated with hypoacetylation (de-acetylated state), and di- or trimethylation of the nineth lysine residue on H3 (H3K9me2 or H3K9me3). The open state, euchromatin, is associated with acetylated H3 and H4, and di- or tri-methylation of the fourth lysine residue of H3 (H3K4me2 or H3K4me3; Arney and Fisher, 2004). Thus, histone methylation, depending on the target of methylation, can result in a change in expression in either direction. There can be coordinated regulation between histone modifications and other epigenetic mechanisms, including DNA methylation (Jin et al., 2011).

**FIGURE 1:**
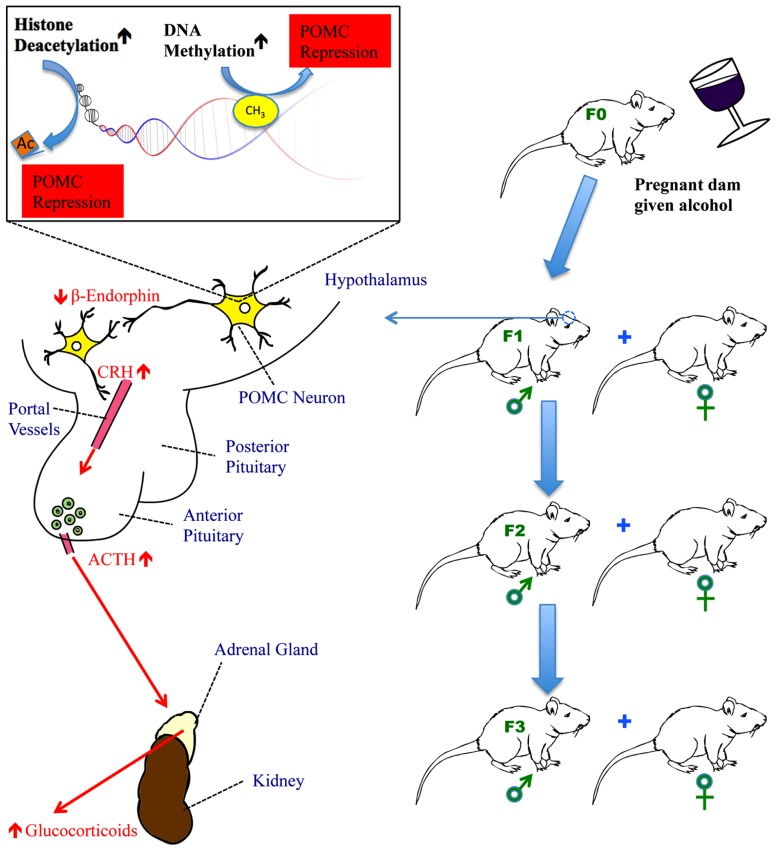
**The impact of fetal alcohol exposure on the hypothalamic-pituitary-adrenal (HPA) axis**. Fetal alcohol exposure increases DNA methyltransferase activity to methylate CpGs in the promoter of the POMC gene in the hypothalamus and activates histone deacetylases to remove acetyl groups from histones in the proximity of the POMC gene. Both methylation and deacetylation lead to the repression of POMC gene transcription. This results in reduced production of β-endorphin, a peptide derived from POMC gene, in the hypothalamus. Reduced β-endorphin production disrupts the normal feedback regulation of the HPA axis causing hyper-response of CRH following stress and subsequently, increased secretion of ACTH from the anterior pituitary and glucocorticoids (corticosterone in rats and cortisol in humans) from the adrenal gland. In fetal alcohol-exposed rats, the HPA axis is dysregulated resulting in hyperstress-response, mental dysfunction, and an impaired immune system. POMC gene hypermethylation and its subsequent impact on the HPA axis persist for a minimum of three generations of progeny, carried through the male germline.

Histone marks have been linked to alcohol consumption (Pal-Bhadra et al., 2007; Pandey et al., 2008). Alcohol causes histone marks to occur in specific genomic locations (Kim and Shukla, 2006). Usually in alcohol studies this has been H3K4me3 (Histone 3 Lysine 4 residue, trimethylated) and acetylation/deacetylation of H3 and H4 (Ponomarev, 2013). Govorko et al. (2012) found that histone deacetylation occurred in fetal rat pups exposed to ethanol, leading to decreased expression of POMC, which partly explains the subsequent disruption of the HPA axis and FASD symptoms observed. See **Figure 1** for a summary of current knowledge.

A number of studies into the impact of ethanol on development have been conducted using cell culture models. Cardiac development was affected by ethanol using cardiac progenitor cells. Under ethanol exposure, it was found that heart development was significantly affected, and H3 acetylation ensued. The effect was dose dependent, leading to greater gene expression changes in higher ethanol doses (Zhong et al., 2010). In fetal neuronal stem cell neurospheres, ethanol was observed to remove histone methylation marks from promoters (either H3K4me3 and H3K27me3, or both) across 20 candidate genes playing roles in processes such as the regulation of neural stem cell biology, and neural patterning (Veazey et al., 2013). Ethanol also impacted the ES-like bivalent signature of the cells (Veazey et al., 2013). Interestingly, for most genes transcriptional control was not changed by the change in histone modifications (Veazey et al., 2013).

By itself, studies of H3K4me3 methylation in relation to alcohol exposure have been found to not be a good predictor of expression (Ponomarev, 2013). Some recent findings have suggested that global chromatin modifications in relation to drug exposure tend to be transient, and revert back to “normal” states within hours to days after the toxicant is removed suggesting that global changes may not be informative (Nestler, 2014). Though transient in some studies, if damage from aberrant methylation occurs at critical windows such as during fetal development when fundamental structures are being laid down, then at least some of the effects could be permanent.

Epigenetic modifications such as those described above have been linked to many diseases, including major mental disorders such as depression, schizophrenia, bipolar disorder (“manic-depression”), and addiction (Jaenisch and Bird, 2003; Eger et al., 2004; Hsieh and Gage, 2005; Sharma et al., 2008; Maze and Nestler, 2011). Haley et al. (2006) found that the HPA stress axis in infants was impacted from fetal alcohol exposure, likely due to epigenetic changes during gastrulation. Given the links between the HPA axis, FASD and mental disorders, understanding the dysregulation of POMC through epigenetic mechanisms could lead to a better understanding of these diseases and new avenues of treatment.

### MicroRNAs

MicroRNAs (miRNAs) are a class of non-coding RNAs. They are roughly 22nt in length and function primarily by targeting the 3′ untranslated region (UTR) of transcripts, leading to their downregulation. MiRNAs increasingly have been found to be important post-transcriptional gene regulators involved in many biological processes widely across animals and most kingdoms (Reviewed in Berezikov, 2011). The effects of microRNAs in fetal alcohol exposure can occur both at the genetic level and at the level of epigenetics. MiRNA expression has been observed to vary between individuals, leading to changes in predisposition to, and severity of, diseases (Chu et al., 2012; Lukiw, 2013). In some cases, single nucleotide polymorphisms (SNPs) in the genes coding for miRNAs impact their expression and function (Sun et al., 2009). These individual differences could lead to significant differences in the consequences to offspring under maternal alcohol consumption. Alternatively, microRNA expression can be modified through epigenetic mechanisms by ethanol (Miranda, 2012), and, as microRNAs interact directly with epigenetic machinery at multiple levels (reviewed in Singh and Campbell, 2013), it seems likely that maternal ethanol consumption could also affect DNA methylation and histone acetylation/deacetylation via microRNAs. As miRNAs have been implicated in FASD models in rodent studies, particularly in neural development (Wang et al., 2009; Miranda, 2012), changes to miRNA expression likely have a significant role in FASD development in the children of women who abuse alcohol.

There is also evidence of miRNA interaction with the HPA stress axis. Recently, it was demonstrated in mice that paternal stress could impact the HPA stress axis, via epigenetic mechanisms acting on DNA in sperm to transmit heritable changes (Rodgers et al., 2013). Among the microRNAs in the list of those impacted by paternal stress which interact with the HPA stress axis, the expression of miR-29c and miR-204 were found to change in expression due to alcohol exposure in adult rat prefrontal cortex (PFC; Tapocik et al., 2013), whereas miR-29c and -30a were found to be altered in fetal mouse brains during exposure to maternal ethanol (Wang et al., 2009), suggesting that these microRNAs may impact HPA axis development during fetal alcohol exposure.

## TRANSGENERATIONAL EPIGENETIC INHERITANCE OF FASD SYMPTOMS

### HERITABLE EPIGENETIC MODIFICATIONS PLAY A ROLE IN MANY DISEASES

Transgenerational epigenetic inheritance, changes carried through the germline (Guerrero-Bosagna et al., 2010; Thornburg et al., 2010), provides an explanation for questions that molecular biologists have not been able to answer through genetics alone. Heritable epigenetic modifications play a key role in some human diseases. Several syndromes in the literature include Angelman, Prader-Willi, and Beckwith-Wiedemann syndrome. These are linked to heritable changes, such as deletions, in specific chromosome regions that undergo genomic imprinting (Adams, 2008). In imprinting, parental differences in epigenetic modifications of specific genes occur in sperm and ova, resulting in parent-specific gene expression. When the contribution to a gene from one parent is silenced through epigenetic mechanisms, deleting the copy from the other parent results in loss of the gene function.

### TRANSGENERATIONAL EPIGENETIC MODIFICATIONS ARE RARE

Transgenerational epigenetic marks on genes are less common than transient marks as most epigenetic signatures are typically lost during gametogenesis. In some cases, certain marks may be retained due to a bias in removal (Morison and Reeve, 1998). Some genes continue to show parental methylation of promoter regions (Borgel et al., 2010). Many epigenetic marks are removed at meiosis (Bond and Finnegan, 2007), and the DNA is methylated again by during early development, often to mark cell type specificity (Shi and Wu, 2009), as there are over 200 different cell types in the body (Alberts et al., 2002).

Prior to fertilization, the male gamete carries the father’s germline epigenetic signature. However, when fertilization occurs, most of the male’s epigenetic marks are lost. Late in the production of sperm, protamines condense the paternal DNA, protecting it and replacing most histones. However, not all histones are replaced; some histones that remain will keep their prior epigenetic marks (Hammoud et al., 2009; Brykczynska et al., 2010). During the interaction of sperm and egg, after the nuclear membranes fuse and the sperm DNA is deposited, the protamines are lost, replaced with histones from the egg. The DNA from the male enters a more open state- most of the replacing histones are acetylated and the much of the methylation present in the DNA is removed (Oswald et al., 2000; Fulka et al., 2004). Interestingly, some regions seem to be protected from demethylation including imprinted genes (Li, 2002). Following fusion, maternal enzymes re-methylate significant regions of the sperm DNA.

Transgenerational epigenetic effects due to exposure to hazardous chemicals have been documented for 4 or more generations (Anway et al., 2005). To be considered a transgenerational epigenetic effect, the effect must persist for multiple generations. The number of generations required to demonstrate a transgenerational epigenetic effect differs, depending on whether the effect is maternally or paternally transmitted. For maternal transmission, three affected generations of offspring are required to demonstrate a transgenerational epigenetic effect. This is because the fetus (F1) inside the mother (F0) is developing gonads (which give rise to the F2 generation), so a purely environmental effect could in theory directly impact the grandchild through alterations to the gonads of F1, leading to a multigenerational effect, but not demonstrating heritable epigenetic transmission. Therefore, to demonstrate persistence of the effect, three generations (F3) of progeny must be impacted. For paternal transmission, the minimum number of impacted generations is two; that is the grandchild of the founder. Sperm could be directly impacted by an environmental agent, leading to an impact on the F1 offspring, but it would require an epigenetic mechanism to then transmit this effect to the F2 generation (Jirtle and Skinner, 2007).

### TRANSGENERATIONAL EPIGENETIC MODIFICATIONS CAUSED BY MATERNAL ALCOHOL CONSUMPTION CAN PROCEED THROUGH THE MALE GERMLINE

There are a number of multigenerational demonstrations of the heritability of alcohol-related disorders in the literature. In a recent study of Native American women who abused alcohol, F2 generation offspring (that is the grandchildren) of an alcohol-abusing woman have a higher tendency to show FAS than those who do F2 progeny of control women (Kvigne et al., 2008). Recently, Govorko et al. (2012) showed that following *in utero* exposure to ethanol, regulatory regions of POMC in the hypothalamus of rats undergo epigenetic modifications: altered histone marks and DNA methylation of the proximal promoter. In addition, histone modifying HDACs and DNA methyltransferases (DNMTs) were shown to be impacted, suggesting a causal relationship between alcohol and epigenetic changes. As a result, POMC neurons are impacted across at least three generations, perturbing the expression of key POMC-derived peptides such as β-endorphin, and affecting the production of its downstream messenger corticosterone leading to dysregulation of the HPA axis and an elevated response to stress in the adult offspring. This was the first demonstration of a true transgenerational epigenetic effect for prenatal alcohol exposure. Interestingly, Govorko et al. (2012) were able to reverse this effect through HDAC and DNA methylation inhibitors, providing additional support for their conclusion.

There is also evidence that hypomethylation occurs in the sperm of alcoholic men (Ouko et al., 2009). Transmission of the effects of alcohol through the male germline has precedents in the literature for induction of symptoms like those found in FASD. These include mental impairment, cardiac defects, low birth weight, and hyperactivity, compared to controls, as assessed in human epidemiological studies and backed by animal studies (Abel, 2004). This supports the findings of Govorko and colleagues that factors that impact POMC and subsequently affect the HPA axis and FASD, can be transmitted by males through the germline.

## SUMMARY AND FUTURE DIRECTIONS

FASD is caused by a complex interaction of genes and environment, and is regulated by both parental and fetal genes. Some symptoms of FASD are caused by decreased expression of POMC, and it’s peptide product, β-endorphin, important in the HPA stress axis regulation. Recent studies by Govorko et al. (2012) have elucidated that POMC epigenetic changes are transmitted through pups in the male germline descended from fetal alcohol exposed animals for several generations. It is currently unknown how this occurs, as direct changes to the enzymes involved in methylation and deacetylation should also impact female progeny. It is speculated that the non-pairing region of the Y chromosome, which is euchromatic, may be partly protected from demethylation and could carry the epigenetic modifications to future male progeny.

The reversibility of the POMC system defect that Govorko and colleagues demonstrated via the modulation of the components of the epigenetic machinery may have therapeutic potential. Histone deacetylase inhibitors have proven to be effective in reducing some symptoms of alcohol damage. These act by preventing HDACs from removing acetyl groups from the tails of histones, and ultimately maintain a potentially transcriptionally active state. Rat studies have shown that this can improve the symptoms of FASD (Govorko et al., 2012), and also can function to prevent tolerance and withdrawal in adult rats (Sakharkar et al., 2012).

Choline influences SAM levels, and choline deficiency during development phenotypically mimics folate deficiency (Zeisel, 2004, 2006). Choline chloride supplementation has been successful in reducing the impact of maternal alcohol consumption on developing fetuses (Thomas et al., 2007, 2010; Bekdash et al., 2013). The use of choline and HDAC or DNMT inhibiting supplements to mitigate FAS symptoms in rats suggests that supplementation could assist at-risk populations during pregnancy, though more studies need to be done in this area. In addition, developments in the understanding of epigenetic regulation of POMC may suggest additional treatment strategies to reduce symptoms of the illness related to the HPA stress axis in adults, and given the links to other disorders, such as cancer and mental disorders, the effects of this research could be far-reaching. The future is promising.

## Conflict of Interest Statement

The authors declare that the research was conducted in the absence of any commercial or financial relationships that could be construed as a potential conflict of interest.
